# Identification of a Novel Ichthyic Parvovirus in Marine Species in Hainan Island, China

**DOI:** 10.3389/fmicb.2019.02815

**Published:** 2019-12-05

**Authors:** Jiang Du, Wenqi Wang, Jasper Fuk-Woo Chan, Gaoyu Wang, Yi Huang, Yufang Yi, Zheng Zhu, Ruoyan Peng, Xiaoyuan Hu, Yue Wu, Jifeng Zeng, Jiping Zheng, Xiuji Cui, Lina Niu, Wei Zhao, Gang Lu, Kwok-Yung Yuen, Feifei Yin

**Affiliations:** ^1^Hainan Medical University-The University of Hong Kong Joint Laboratory of Tropical Infectious Diseases, Hainan Medical University, Haikou, China; ^2^Key Laboratory of Tropical Translational Medicine of Ministry of Education, Hainan Medical University, Haikou, China; ^3^Department of Pathogen Biology, Hainan Medical University, Haikou, China; ^4^State Key Laboratory of Emerging Infectious Diseases, The University of Hong Kong, Pokfulam, Hong Kong; ^5^Department of Microbiology, The University of Hong Kong, Pokfulam, Hong Kong; ^6^Carol Yu Centre for Infection, The University of Hong Kong, Pokfulam, Hong Kong; ^7^Key Laboratory of Tropical Animal Breeding and Epidemic Disease Research of Hainan Province, Hainan University, Haikou, China; ^8^Key Laboratory of Tropical Biological Resources of Ministry of Education, Haikou, China; ^9^Hainan Key Laboratory for Sustainable Utilization of Tropical Bioresources, Hainan University, Haikou, China

**Keywords:** *Parvoviridae*, novel virus, *Chaphamaparvovirus*, crocodile, tilapia

## Abstract

Parvoviruses are a diverse group of viruses that are capable of infecting a wide range of animals. In this study, we report the discovery of a novel parvovirus, tilapia parvovirus HMU-HKU, in the fecal samples of crocodiles and intestines of tilapia in Hainan Province, China. The novel parvovirus was firstly identified from crocodiles fed with tilapia using next-generation sequencing (NGS). Screening studies revealed that the prevalence of the novel parvovirus in crocodile feces samples fed on tilapia (75–86%) was apparently higher than that in crocodiles fed with chicken (4%). Further studies revealed that the prevalence of the novel parvovirus in tilapia feces samples collected at four areas in Hainan Province was between 40 and 90%. Four stains of the novel parvovirus were identified in this study based on sequence analyses of NS1 and all the four strains were found in tilapia in contrast only two of them were detected in crocodile feces. The nearly full-length genome sequence of the tilapia parvovirus HMU-HKU-1 was determined and showed less than 45.50 and 40.38% amino acid identity with other members of *Parvoviridae* in NS1 and VP1 genes, respectively. Phylogenetic analysis based on the complete helicase domain amino acid sequences showed that the tilapia parvovirus HMU-HKU-1 formed a relatively independent branch in the newly proposed genus *Chaphamaparvovirus* in the subfamily *Hamaparvovirinae* according to the ICTV’s most recent taxonomic criteria for *Parvoviridae* classification. Tilapia parvovirus HMU-HKU-1 likely represented a new species within the new genus *Chaphamaparvovirus*. The identification of tilapia parvovirus HMU-HKU provides further insight into the viral and genetic diversity of parvoviruses and its infections in tilapia populations need to be evaluated in terms of pathogenicity and production losses in tilapia farming.

## Introduction

Members of the family *Parvoviridae* are non-enveloped, non-segmented, single-stranded DNA viruses with an average genome size of 4000–6000 nucleotides (Mietzsch et al., 2019). The family *Parvoviridae* currently comprises two subfamilies, namely, *Parvovirinae* and *Densovirinae*. There are currently 59 species in the subfamily *Parvovirinae*, divided into eight genera: *Amdoparvovirus*, *Aveparvovirus*, *Bocaparvovirus*, *Copiparvovirus*, *Dependoparvovirus*, *Erythroparvovirus, Protoparvovirus*, and *Tetraparvovirus* (Cotmore et al., 2019). There are 21 species in the *Densovirinae* subfamily, divided into five genera: *Ambidensovirus, Brevidensovirus, Hepandensovirus, Iteradensovirus*, and *Penstyldensovirus*. Recently, the novel subfamily *Hamaparvovirinae* was additionally proposed to be included into *Parvoviridae*. This new subfamily comprises of the genera *Chaphamaparvovirus*, the recently discovered *Ichthamaparvovirus*, and the known genera *Brevidensovirus, Hepandensovirus*, and *Penstyldensovirus*. *Chaphamaparvovirus* was a novel genus of parvoviruses which did not belong to the subfamilies *Parvovirinae* nor *Densovirinae* (Palinski et al., 2016), while the genus *Ichthamaparvovirus* contained one species, namely, *Syngnathus scovelli* chapparvovirus (Pénzes et al., 2019).

Many members of *Parvoviridae* are known human and/or animal pathogens. The viruses in the subfamily *Parvovirinae* only infect vertebrates and may cause disease in human (Qiu et al., 2017). The most well-known human pathogen in this subfamily of viruses is parvovirus B19, a member of the genus *Erythroparvovirus* (Heegaard and Brown, 2002). Parvovirus B19 may cause hydrops fetalis in fetuses, erythema infectiosum in children, arthritis in adults, and aplastic crisis in patients with hemoglobinopathies (Xu et al., 2019). Human bocavirus (HBoV) genotypes 1–4 in the genus *Bocaparvovirus* are recently discovered human pathogens which may be associated with respiratory tract infections, diarrhea, and acute flaccid paralysis, especially in children (Arthur et al., 2009; Shen et al., 2015). Other members of the subfamily *Parvovirinae* may cause acute hemorrhagic enteritis, gastroenteritis, myocarditis, leukopenia, and fetal demise in domesticated animals, which is associated with a high mortality rate and severe economic loss (Decaro and Buonavoglia, 2012; Alarcon et al., 2013; Ni et al., 2014; Schirtzinger et al., 2015; Cotmore et al., 2019).

Members of *Parvovirinae* are found in many different animal species, including bats, rodents, chipmunks, bovines, and non-human primates, and the infected animals may have asymptomatic or minimally symptomatic infections (Green et al., 2000; Harbison et al., 2009; Chen et al., 2010; Lau et al., 2016; De Souza et al., 2018; Finoketti et al., 2019). Cross-species transmissions of *Parvovirinae* have been reported (Allison et al., 2013). For example, canine parvovirus two first appeared in 1978, is primarily a dog pathogen that originated from a close relative of a cat pathogen through intermediate hosts (Parker et al., 2001; Zhong et al., 2014). In aquafarming, densoviruses can cause major losses to banana shrimp cultivation (Phuthaworn et al., 2016). However, little is known about the role of parvovirus infections in other forms of aquafarming. In this study, using next-generation sequencing (NGS), we discovered a novel parvovirus, tilapia parvovirus HMU-HKU, in the fecal samples of crocodiles and intestines of tilapia in Hainan Province, China. Tilapia parvovirus HMU-HKU is phylogenetically distinct from all known parvoviruses. The nearly complete genome sequence of the novel tilapia parvovirus HMU-HKU was determined and characterized.

## Materials and Methods

### Fecal Samples of Crocodiles and Fish

**FIGURE 1:**
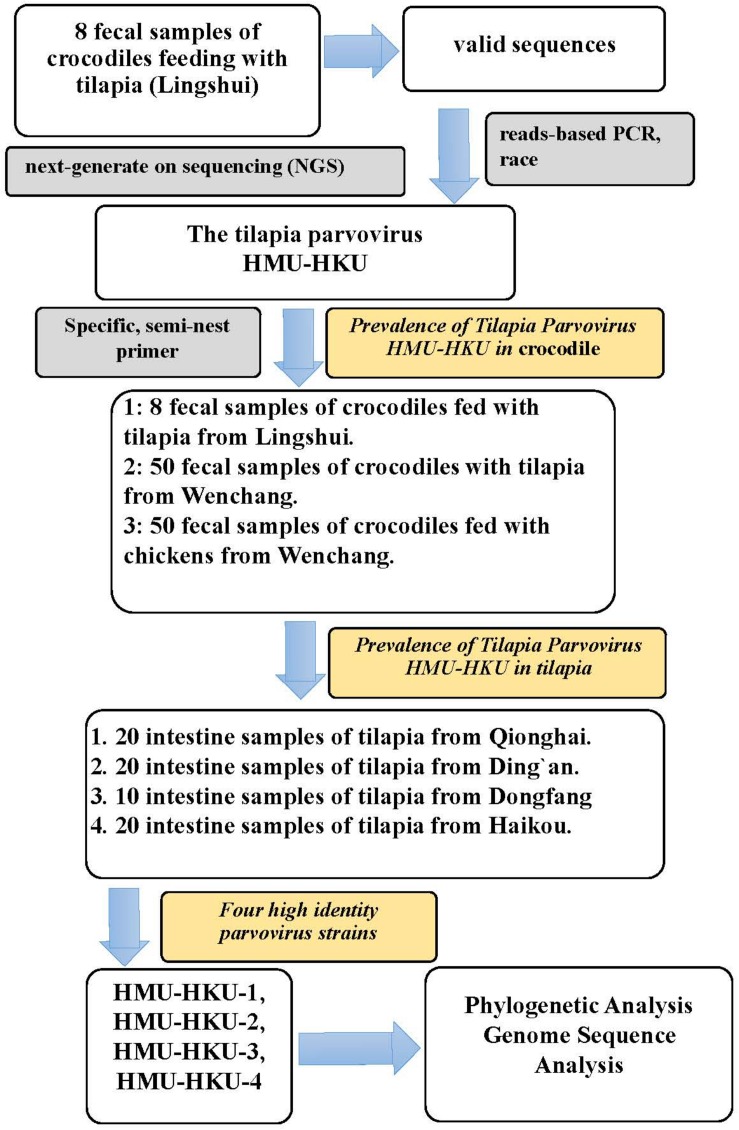
Flow chart of the study’s procedures. The discovery of the novel tilapia parvovirus HMU-HKU and the prevalence rate of tilapia parvovirus HMU-HKU among crocodiles and tilapia in different regions of Hainan.

The sampling procedures were approved by the Ethics Committee of the Hainan Medical University. A total of 108 fecal samples were collected from 108 different crocodiles (*Crocodylus siamensis*) which were fed with tilapia or chicken at three crocodile breeding sites located in the counties/cities of Wenchang and Lingshui of Hainan Province (Figure 1). Following the discovery of the tilapia parvovirus HMU-HKU, a total of 70 intestine samples were additionally collected from 70 different tilapia (*Oreochromis niloticus*) at four tilapia breeding sites located in four counties/cities (Ding’an, Dongfang, Haikou, and Qionghai) of Hainan Province, China (Supplementary Figure 1). The samples were immersed in maintenance medium in virus-sampling tubes (Yocon/Beijing, China, which is composed of Hank’s, gentamicin, fungal antibiotics, bovine serum albumin) and were transported to the laboratory within 24 h using cold chain transportation and stored at −80°C. The tilapia species was determined morphologically and confirmed by DNA sequence analysis of mitochondrial cytochrome b (Kiril’chik et al., 1995).

### Viral Nucleic Acid Library Construction and NGS

Eight fecal samples from crocodiles fed with tilapia in Lingshui County were pooled and processed using a virus-particle-protected, nucleic acid purification protocol and nucleic acid library preparation method, as described previously (Wu et al., 2018). Amplified viral nucleic acid libraries were analyzed on an Illumina HiSeq2500 sequencer, for a single read of 100 bp in length. The raw sequence reads were filtered using previously described criteria to obtain valid sequences (Yang et al., 2011).

### Taxonomic Assignment

Sequence-similarity-based taxonomic assignments were conducted as described previously (Yang et al., 2011). Briefly, each read was evaluated for viral origin by conducting alignments in the National Center for Biotechnology Information (NCBI) non-redundant nucleotide database (NT), and protein database (NR), using BLASTn and BLASTx (-e: Expected value < 10^–5^, -F: Filter query sequence, default = T). Taxonomies of aligned reads with the best BLAST scores (*E*-value < 10^–5^) from all lanes were parsed and exported using the MEGAN 6- MEtaGenome Analyzer (Tamura et al., 2013).

### Genome Sequencing of Parvoviruses

The molecular clues provided by metagenomic analyses were used to classify sequence reads into a virus family or genus using MEGAN 6. Viral nucleotide (including both viral genome DNAs and viral mRNAs) was isolated using a QIAmp MinElute Virus Spin Kit (Qiagen/United States) from the specimens and used as the template for genome sequencing. Gene-specific primers were designed based on representative identified reads of the novel parvovirus to cover the partial genomes by PCR amplification and Sanger sequencing. The remaining genomic sequences were determined by genome walking (Takara/Japan), 5’-race system version 2.0 combo (Invitrogen/United States) and 3’-Full RACE Core Set with PrimeScript RTase (Takara/Japan).

### Prevalence of Parvovirus Infection Among Sampled Fish and Crocodiles

Using the complete genomic sequences of the viruses obtained by the ends amplification, we designed specific, semi-nest primers targeting the NS1 gene for PCR to screen for parvoviruses in the fecal samples from crocodiles and intestine samples of tilapia. PCRs were performed using 2 × Taq PCR Mastermix (Tiangen, China), and the first round PCR was primed with outer primers (TParv-F:5′-TAATAAAGCGGGCGTGGAACA-3′ and TParv-R:5′-CTATTACTCAATGCTGCTCGTTCA-3′) and the second round PCR was primed with inner primers (TParv-Fn:5′-ATTACAGAGAAGTGACCGTTTTAG-3′ and TParv-R). 2 μl of the first round PCR product was used as the template for the second round of PCR. The thermal cycling conditions for both PCRs were 94°C for 5 min, followed by 35 cycles of 94°C for 30 s, 57°C for 30 s, 72°C for 45 s, and a final elongation step at 72°C for 10 min. Finally, the PCR products were analyzed on 1.5% agarose gel electrophoresis ultraviolet imaging. Positive samples were determined with 872 bp amplified products. Approximately 30% of the PCR products were randomly selected for Sanger sequencing for confirmation.

### Genome Annotation

The nucleotide sequences of the genomes and the amino acid sequences of the ORFs were deduced by comparing the sequences with those of other parvoviruses. Conserved protein families and domains were predicted using Pfam and InterProScan 5^1^. Routine sequence alignments were performed using Clustal Omega, Needle^2^ (Zhu et al., 2016).

### Phylogenetic Analysis

MEGA6.0 was used to align nucleotide sequences and deduce amino acid sequences, using the MUSCLE package and default parameters. The phylogenetic tree was constructed with the maximum-likelihood method. The substitution models rtREV with Freqs(+F) model were carried out by model selection function of MEGA6.0 with 1000 bootstrap replicates. Amino acid pairwise alignment between Tilapia parvovirus HMU-HKU and other members of *Parvoviridae* were performed with NCBI Basic Local Alignment Search Tool. The p-distances were calculated using the “Compute Pariwise Distances” calculation in MEGA6 (Supplementary Table 1).

### Nucleotide Sequence Accession Numbers

The near complete genome sequence of tilapia parvovirus HMU-HKU (HMU-HKU-1) and three complete ORFs of NS1 (HMU-HKU-2, HMU-HKU-3, and HMU-HKU-4) were deposited to GenBank under accession numbers MN162688 to MN162691. The Illumina HiSeq2500 sequence data of crocodilian feces samples were deposited into the NCBI sequence reads archive (SRA) under accession number PRJNA543153.

## Results

### Discovery of a Novel Parvovirus in Crocodiles by NGS

To identify novel viruses in crocodiles in Hainan, we first collected eight fecal samples from eight different crocodiles in a crocodile breeding site in the Lingshui County in the Southern part of Hainan (Supplementary Figure 1). The eight samples were combined into one pool for NGS. A total of 3.7 GB of nucleotide data (38,225,149 valid reads, 100 bp in length) were obtained. The reads that were classified as cellular organisms (bacteria, archaea, and eukaryotes) and reads with no significant similarities to any amino acid amino acid sequence in the NR database were removed. The remaining 197,800 reads were best-matched with viral proteins from the NR database (approximately 0.52% of the total sequence reads). There were 620 *Parvovirinae*-associated reads in the lane. They were assembled as one contig for 902 bp genome fragment.

### Genome Structure Analysis of HMU-HKU Tilapia Parvovirus

**FIGURE 2:**
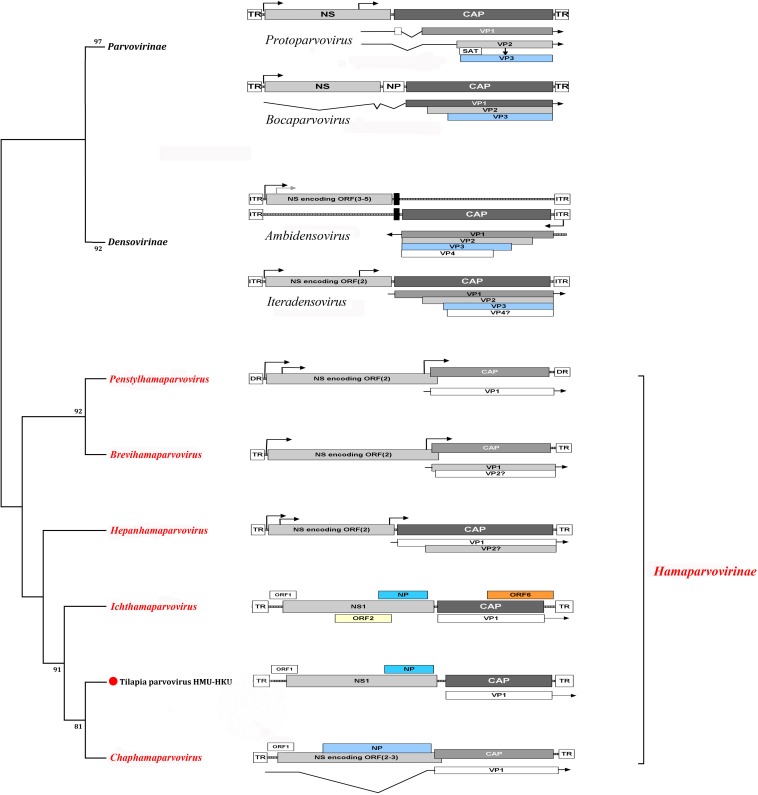
Cladogram of subfamilies *Hamaparvovirinae*, *Parvovirinae*, and *Densovirinae*. The genome organizations of members of the representative genera of the three subfamilies are shown. The novel tilapia parvovirus HMU-HKU-1 discovered in this study was labeled with a red circle (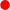). The genera and subfamilies described in the newly proposed ICTV parvovirus taxonomic classification were highlighted in red.

Based on the NGS-generated fragment sequences, primers were designed to obtain the intervening portions of the genome. The terminal sequences were then acquired using a 5′- and 3′-RACE method. The nearly complete genome of the novel tilapia parvovirus HMU-HKU-1 was 3566 bp in length (Figure 2), with a G+C content of 43.89%. A putative ORF1 (nt 24–350) was found before the putative NS1. NS1 (nt 269–2125) was 1,857 bp in length and encoded a 618-amino acid non-structural protein. The NS1 in tilapia parvovirus HMU-HKU1 had less than 45.5% amino acid identity with the NS1’s of other known members of *Parvoviridae*. A putative nucleoprotein (NP) was identified to be overlapping with the NS1 at nt 1428–2039. This putative NP shared only 33.99% amino acid identity with that of simian parvo-like virus 3 (accession number: KT961660). The VP1 ORF was 1,260 bp in length and encoded a putative 419-amino acid capsid protein VP1. The capsid protein VP1 had less than 40.38% amino acid identity with that of other known members of *Parvoviridae*. Based on these findings, the predicted genome structure of the novel tilapia parvovirus HMU-HKU-1 is more similar to that of members of *Chaphamaparvovirus* than those of the other members of *Parvoviridae*.

At the nucleic acid level, the novel tilapia parvovirus HMU-HKU-1 genome sequence was barely comparable with other known parvoviruses in the NCBI nucleotide database. Conserved polyadenylation signals were absent, downstream of the putative NS1 and the VP protein-coding regions (Arthur et al., 2009). In the putative NS1 protein, the following motifs were present: a Walker loop motif, conserved domains for viral replication, a ^380^GASSSGKS^387^ [GxxxxGK(T/S)] domain (Walker et al., 1982), a catalytic domain for rolling circle replication, and a ^45^HYHVLV^50^ domain [HUHUUU, U being hydrophobic amino acid] (Ilyina and Koonin, 1992).

### Phylogenetic Analysis

**FIGURE 3:**
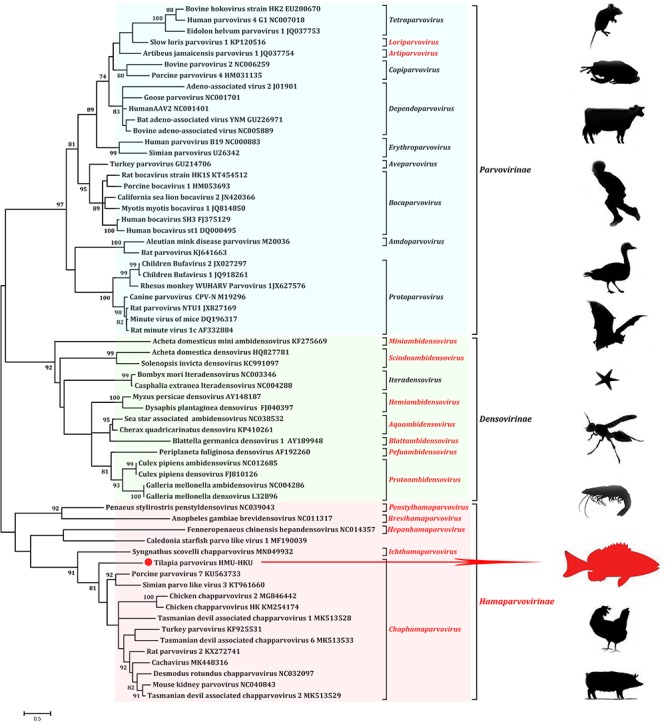
Phylogenetic tree showing the relatedness between the novel tilapia parvovirus HMU-HKU-1 with other members of *Parvoviridae* based on their complete helicase domain amino acid sequences. The novel tilapia parvovirus HMU-HKU-1 discovered in this study was labeled with a red circle (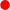). The genera and subfamilies described in the newly proposed ICTV parvovirus taxonomic classification were highlighted in red.

Phylogenetic analysis of the novel tilapia parvovirus HMU-HKU-1 was conducted with 62 reference genome sequences of viruses in the family of *Parvoviridae* available in GenBank (accessed on 1st August 2019). Evolutionary trees were constructed for the complete protein sequences of helicase domain (Figure 3). The topologies of the tree showed that the tilapia parvovirus HMU-HKU-1 formed a relatively independent branch in the newly proposed genus *Chaphamaparvovirus* in the subfamily *Hamaparvovirinae*. The *Syngnathus scovelli* ChVP, another fish parvovirus and the only member of the novel proposed genus *Ichthamaparvovirus*, clustered with *Chaphamaparvovirus* in the same branch, and represented a deeper rooted branch in the subfamily *Hamaparvovirinae* (Pénzes et al., 2019).

Pairwise comparisons were performed for the nucleotide and amino acid sequences of tilapia parvovirus HMU-HKU-1 with other parvoviruses. The results showed that the genome of tilapia parvovirus HMU-HKU-1 shared less than 8% similarity with other members of *Parvoviridae* at nucleotide level and was barely comparable with other known parvoviruses. At the amino acid level, tilapia parvovirus HMU-HKU-1 also exhibited low level of similarity with other members of *Parvoviridae.* The NS1 and VP1 of tilapia parvovirus HMU-HKU-1 exhibited ≤45.50 and ≤40.38% amino acid identity, respectively, with those of *Chaphamaparvovirus*. The NS1 of tilapia parvovirus HMU-HKU-1 exhibited 31.37% amino acid identity with that of the recently reported *Syngnathus scovelli* ChPV and exhibited the highest identity of 45.50% with that of porcine parvovirus 7 (accession number: KU563733). As the NS1 of tilapia parvovirus HMU-HKU-1 exhibited ≥35% amino acid identity with those of *Chaphamaparvovirus*, this novel virus likely represented a new species within the new genus *Chaphamaparvovirus* according to the ICTV’s most recent taxonomic criteria for *Parvoviridae* classification.

### Prevalence of Tilapia Parvovirus HMU-HKU Among Crocodiles and Tilapia in Different Regions of Hainan

As the eight crocodiles we sampled in Lingshui County were exclusively fed with tilapia, we further investigated the differential prevalence rates of tilapia parvovirus HMU-HKU among crocodiles and tilapia in different regions of Hainan. To do this, we first designed a novel semi-nest PCR assay using specific primers targeting the viral NS1 gene. Next, we collected 50 fecal samples from 50 different crocodiles which were fed exclusively with tilapia, and another 50 fecal samples from 50 different crocodiles which were originally fed with tilapia until 2 weeks before sampling, after which they were exclusively fed with chickens. These two cohorts of crocodiles were bred in two different breeding sites in the Wenchang County at the northeastern part of Hainan. As shown in Supplementary Figure 1 and Table 1, the prevalence rate of tilapia parvovirus HMU-HKU in the crocodiles fed exclusively with tilapia was 86.0% (43/50), which was significantly higher (*P* < 0.001) than the one of the crocodiles fed with chicken (2/50, 4.0%).

**TABLE 1:** The prevalence of parvovirus in the population of tilapia and crocodile.

**Location**	**Number**	**Positive rate**	**Strain**	**Sample type**	**Comment**
Lingshui	8	75%(6/8)	HMU-HKU-1	Feces samples of crocodile	Feeding with tilapia
Wenchang	50	86%(43/50)	HMU-HKU-1,HMU-HKU-3	Feces samples of crocodile	Feeding with tilapia
Wenchang	50	4% (2/50)	HMU-HKU-1	Feces samples of crocodile	Feeding with chicken
Haikou	20	40%(8/20)	HMU-HKU-1, HMU-HKU-4	Feces samples of tilapia	
Ding‘an	20	80%(16/20)	HMU-HKU-1, HMU-HKU-2, HMU-HKU-3	Feces samples of tilapia	
Qionghai	20	90%(18/20)	HMU-HKU-4	Feces samples of tilapia	
Dongfang	10	80%(8/10)	HMU-HKU-1, HMU-HKU-4	Feces samples of tilapia	

**FIGURE 4:**
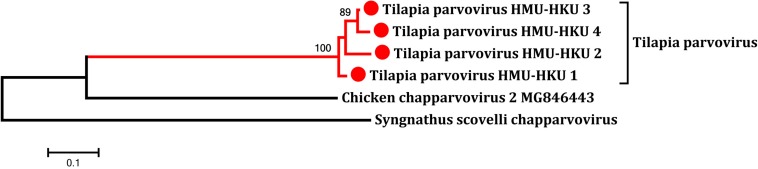
Phylogenetic tree showing the relatedness between the novel tilapia parvoviruses HMU-HKU-1 to 4 with other representative member of *Chaphamaparvovirus* and *Ichthamaparvovirus* in the novel subfamily *Hamaparvovirinae* based on their complete NS1 nucleotide sequences. The novel tilapia parvoviruses HMU-HKU-1 to 4 were labeled with red circles (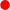).

To determine whether the novel tilapia parvovirus HMU-HKU was also found in the tilapia in other regions of Hainan, we then collected additional intestine samples from tilapia bred in other counties/cities in the province. As shown in Supplementary Figure 1, the virus is highly prevalent among tilapia bred in different locations, including Qionghai (18/20, 90.0%), Ding’an (16/20, 80.0%), Dongfang (8/10, 80%), and Haikou (8/20, 40.0%). The positive NS1 amplicon in feces samples from crocodile and tilapia were sequenced. On the basis of phylogenetic analysis and multiple sequence alignments of nucleotide sequences, there were four high identity parvovirus strains; HMU-HKU-1, HMU-HKU-2, HMU-HKU-3, and HMU-HKU-4, with 92.6–94.9% nucleotide identify with each other. The four strains formed an independent cluster with outgroup *Chaphamaparvovirus* (Figure 4). In multiple amino acid sequence alignments, in the Walker loop motif, [GxxxxGK(T/S)] domain, the first two amino acids are GA in strain 1 and 3, ST in strain 2 and DK in strain 4 (Supplementary Figure 2).

## Discussion

With the advances and availability of virus discovery tools such as highly sensitive PCRs and NGS, an increasingly number of parvoviruses have been discovered in the past few decades. Parvovirus B19, discovered in 1974, was the first identified parvovirus and also the best known human-pathogenic parvovirus (Heegaard and Brown, 2002). HBoVs were first discovered in 2005 from pooled nasopharyngeal aspirates (Guido et al., 2016). Parvovirus 4 was another parvovirus frequently detected in injecting drug users and hemophiliacs which was also first discovered in 2005 (Jones et al., 2005; Sharp et al., 2009). Three novel parvoviruses belonging to the genus *Protoparvovirus* have been identified in human fecal specimens in the past decade, namely, bufavirus in 2012, tusavirus in 2014, and cutavirus in 2016 (Vaisanen et al., 2017). These viruses were predominantly detected in children with diarrhea. Bufavirus has also been found in other mammalian species, including shrews, bats, and non-human primates, and cutavirus has also been detected in malignant skin tissues of patients with cutaneous T-cell lymphoma (Phan et al., 2016; Yang et al., 2016) Canine parvovirus and feline panleukopenia virus are important pathogens of dogs and cats, respectively (Parker et al., 2001). Porcine parvoviruses, including ungulate protoparvovirus 1, ungulate tetraparvovirus 2 and 3, ungulate copiparvovirus 2, ungulate bocaparvovirus 2, 3, 4, and 5, and porcine parvovirus 6, cause porcine reproductive failure in pigs (Schirtzinger et al., 2015). Duck-origin goose parvovirus is a causative agent in beak atrophy and dwarfism syndrome in ducks (Luo et al., 2019). In adult mink, Aleutian disease causes a generalized systemic syndrome, including fur damage and reproductive disorders such as infertility, abortions and reduced litter size (Prieto et al., 2017). Hepatopancreatic parvovirus or *Penaeus monodon* densovirus causes mortality in early larval and postlarval stages of penaeid shrimps and retarded growth in juveniles (Phuthaworn et al., 2016). The examples highlighted the importance of parvoviruses in human and animal health and the associated economic impact. In contrast to mammalian and avian species, little is known about the presence and significance of parvoviruses in aquatic and semiaquatic animals. In this study, we utilized NGS to discover a novel parvovirus, named tilapia parvovirus HMU-HKU, in the fecal samples of crocodiles and tilapia in Hainan, a major hub of aquafarming in China. Interestingly, the fecal samples of the crocodiles fed exclusively with tilapia had a significantly higher positive rate of this virus than the crocodiles that were fed with chickens (86.0 vs. 4.0%, *P* < 0.001). The low positive rate of 4.0% among the crocodiles fed with chicken likely represented residual viruses in their feces as they were fed with tilapia until 2 weeks before sampling. Therefore, the crocodiles were unlikely the natural reservoirs of tilapia parvovirus HMU-HKU. In many regions of Hainan, tilapias were fed with different fish feeds different, which had a less possibility to be the real hosts of this parvovirus. Indeed, tilapia in different regions of Hainan all showed a high virus positive rate (40.0–90.0%). Taken together, these results suggested that tilapia were likely an important reservoir of the novel tilapia parvovirus HMU-HKU.

Our phylogenetic analysis showed that the novel tilapia parvovirus HMU-HKU was distinct from all other known parvoviruses and should represent a new species in the genus *Chaphamaparvovirus*. According to the International Committee on Taxonomy of Viruses (ICTV), virus taxonomy usually refers to: (i) their host species (or group of host species), (ii) amino acid sequence homology, (iii) genomic structure, and (iv) their defined geographic distributions. Members of the subfamilies *Parvovirinae* and *Densovirinae* mainly infect vertebrates (mammals, birds, and reptiles) and invertebrates (insects, crustaceans, and echinoderms), respectively (Figure 3). In the past few years, a large number of new parvoviruses were reported but they did not conform to the then-established genus demarcation criteria (Cotmore et al., 2014; Cotmore et al., 2019). A new parvovirus taxonomy proposal have been accepted by ICTV recently which split the *Parvoviridae* into three subfamilies^3^. Subfamily *Densovirinae* was divided into eight genera: *Miniambidensovirus*, *Aquambidensovirus*, *Scindoambidensovirus*, *Protoambidensovirus*, *Hemiambi-densovirus*, *Pefuambidensovirus*, *Blattambidensovirus*, and *Iteradensoviru.* Two new genera were added to the subfamily *parvovirinae: Artiparvovirus* and *Loriparvovirus*. Subfamily *Hamaparvovirinae* was comprised of genera *Penstylhamaparvovirus*, b*Brevihamaparvovirus*, h*Hepanham- aparvovirus* and *Ichthamaparvovirus*, together with *Chaphamaparvovirus*. As shown in our phylogenetic tree (Figure 3), tilapia parvovirus HMU-HKU formed a relatively distinct branch within *Chaphamaparvovirus* which was separated from *Ichthamaparvovirus*, h*Hepanhamap- arvovirus, Brevihamaparvovirus* and *Penstylhamaparvovirus* in the deep root of the phylogenetic tree. Notably, the two fish parvoviruses, namely, tilapia parvovirus HMU-HKU-1 and *Syngnathus scovelli* ChPV, belonged to different genera in the new subfamily *Hamaparvovirinae*. However, the topologies of the tree showed that the *Ichthamaparvovirus* and *Chaphamaparvovirus* clustered within the same branch. This suggested that tilapia parvovirus HMU-HKU and *Syngnathus scovelli* ChPV likely shared a common ancestor, but have subsequently independently evolved in their different natural hosts.

The linear genome of *Parvoviridae* generally encodes two major gene cassettes. The NS1, also known as *ns* or *rep* gene, encodes the non-structural proteins which are regulatory proteins indispensable for viral replication. The NS1 of *Parvovirinae*, *Densovirinae*, and *Hamaparvovirinae* encodes up to 1, 5, and 3 proteins, respectively (Mietzsch et al., 2019). The capsid protein of members of *Parvoviridae* is assembled by viral proteins which are encoded by the *cap* gene. The *cap* gene of *Parvovirinae*, *Densovirinae*, and *Hamaparvovirinae* encodes up to 3, 4, and 1 VPs, respectively (Mietzsch et al., 2019). NP, a small regulatory protein, was commonly found in members of *Parvovirinae* and *Hamaparvovirinae.* The tilapia parvovirus HMU-HKU-1 was predicted to encode one small ORF (ORF1), one non-structural protein (NS1), one NP, and one VP. This genome organization is most similar to that of *Chaphamaparvovirus* and suggested that the novel tilapia parvovirus HMU-HKU discovered in this study likely belonged to the genus *Chaphamaparvovirus* in the newly proposed subfamily *Hamaparvovirinae.*

## Conclusion

In this study, we reported the discovery of a novel parvovirus, tilapia parvovirus HMU-HKU, identified in the fecal sample of crocodiles and intestine of tilapia in Hainan Province, China. Our results provided insights into the ecology and evolution of *Parvoviridae* and their hosts among aquatic and semiaquatic animals. The pathogenicity and associated impact on aquafarming of this novel virus should be evaluated in further studies.

## Data Availability Statement

The datasets generated for this study can be found in the GenBank, MN162688–MN162691, NCBI sequence reads archive (SRA), PRJNA543153.

## Ethics Statement

The animal study was reviewed and approved by the Ethics Committee of the Hainan Medical University.

## Author Contributions

JD, WW, and FY designed the study. JD, WW, YY, ZZ, GW, YH, RP, XH, YW, JFZ, JPZ, XC, LN, WZ, and FY collected the specimens and performed the experiments. JD, WW, JC, GL, K-YY, and FY analyzed the data. JD, JC, K-YY, and FY wrote the manuscript. All authors reviewed the manuscript.

## Conflict of Interest

The authors declare that the research was conducted in the absence of any commercial or financial relationships that could be construed as a potential conflict of interest.

## Footnotes

1 http://www.ebi.ac.uk/services/proteins

2 http://www.ebi.ac.uk/Tools/

3 https://talk.ictvonline.org/files/proposals/animal_dna_viruses_and_retroviruses/m/animal_dna_ec_approved/9296
