# Supplementary material for: Identification of a Novel Ichthyic Parvovirus in Marine Species in Hainan Island, China
Source: Front Microbiol. 2019 Dec 5;10:2815. doi: 10.3389/fmicb.2019.02815 (PMC6907010; doi:10.3389/fmicb.2019.02815)
Supplement: Supplementary file 1 [file Data_Sheet_1.docx]

**Supplementary Fig.1. Sampling map.** In total 108 feces samples from crocodile and 70 intestines of tilapia were collected from six areas of Hainan provinces. The size of the pie charts illustrating positive and negative samples is proportional to the numbers of total samples in each area. Positive and negative feces samples of crocodile are labeled with yellow and green, positive and negative intestines of tilapia are labeled with orange and blue, respectively.

## Supplementary Fig.2. Multiple sequence alignments of the complete NS1 amino acid sequences of tilapia parvovirus HMU-HKU1-4. A catalytic domain for rolling circle replication ^45^HYHVLV^50^ and a Walker loop motif ^380^GASSSGKS^387^ were circled.
