# Supplementary figures and images for: Identification of a Novel Ichthyic Parvovirus in Marine Species in Hainan Island, China
Source: Front Microbiol. 2019 Dec 5;10:2815. doi: 10.3389/fmicb.2019.02815 (PMC6907010; doi:10.3389/fmicb.2019.02815)

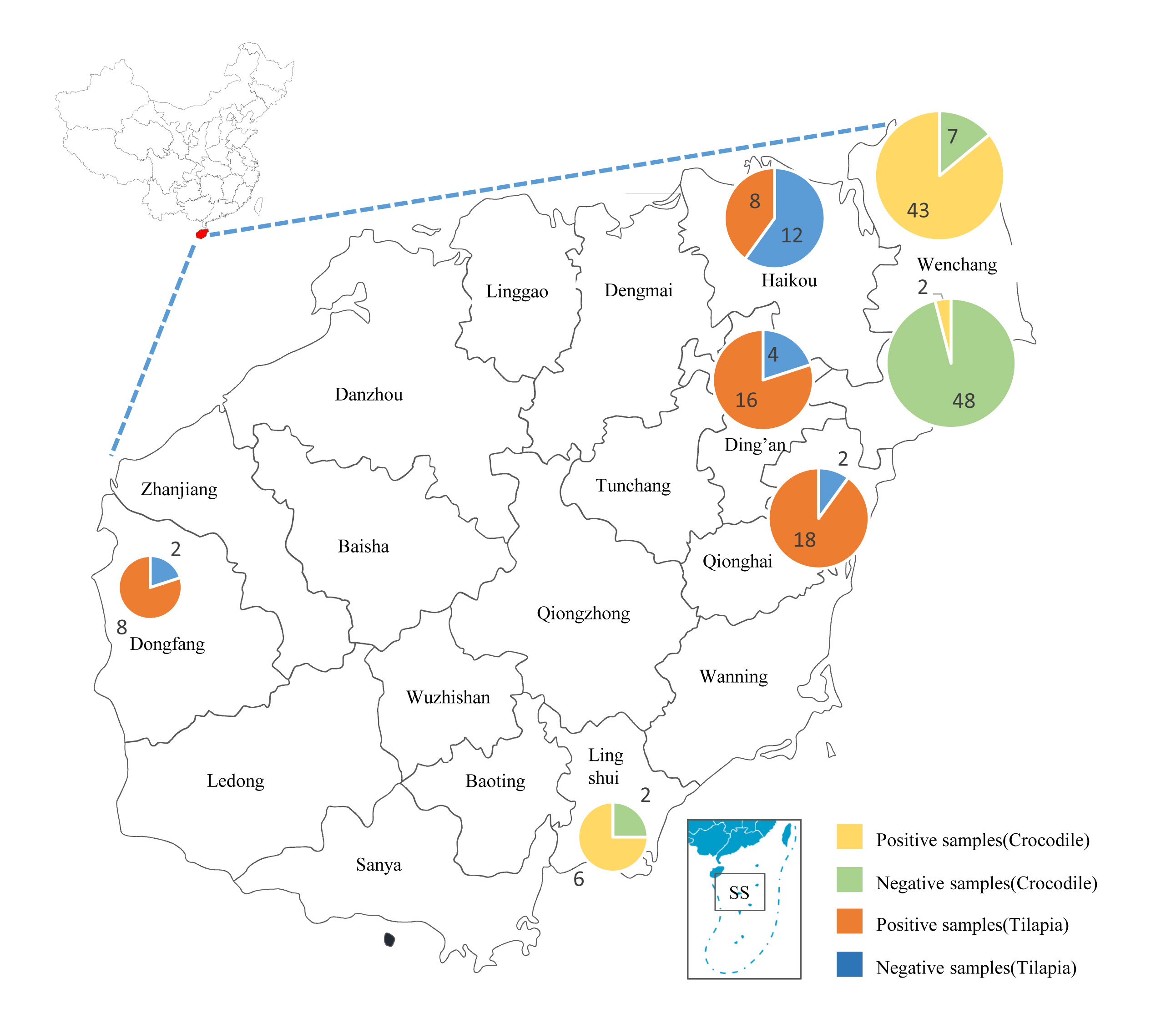

Supplement: Supplementary file 3 [file Image_1.JPEG]

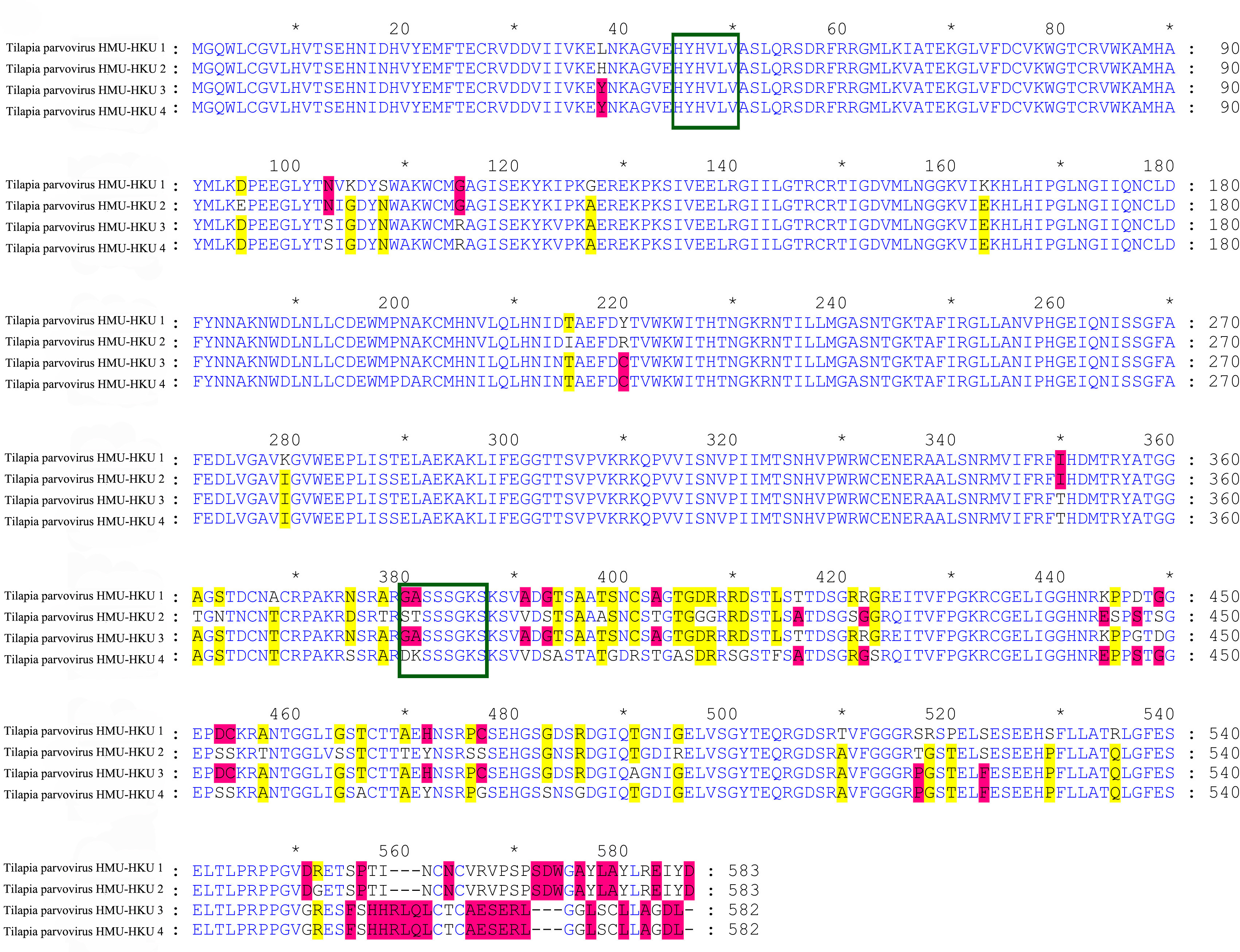

Supplement: Supplementary file 4 [file Image_2.JPEG]
